# Diseases of the Eye

**Published:** 1889-09

**Authors:** 


### Reviews of Books.

---

*Diseases of the Eye.* By GEORGE A. BERRY, M.B., F.R.C.S.Ed. Edinburgh: YOUNG J. PENTLAND. 1889.

This is undoubtedly one of the best, if not the very best, of the books that have yet appeared in the English language on the subject of Ophthalmic Diseases.

It is illustrated by coloured pictures from the brush of Dr. Tatham Thompson of Cardiff,—very well executed, but perhaps some are a little unnatural in colouring. The pictures form, however, a considerable addition to the book, and most of them are very good.

The diseases are described rather from the clinical standpoint than from the pathological; but in ophthalmic work it is particularly difficult to dissociate them. Histological changes are easily seen, especially in diseases of the cornea and iris, in which structures the pathology of inflammation can be most perfectly studied.

The first half of the book, Section I., is concerned with the general diseases affecting the eye and its appendages. The consideration of the affections of the lacrymal passages is very full and satisfactory: the author gives good practical advice in this difficult subject. Diseases of the cornea are well and fully dealt with; and iritis, in its various forms and under its various complications, receives ample consideration. These facts are mentioned to show that the book is one well adapted for the needs of the family practitioner. But it should, at the same time, find a place in every ophthalmic surgeon's library. One of the best chapters is on foreign bodies in the eye. Amblyopia in its different degrees is well worked out. All parts of the clinical portion of the book show wide reading as well as large practical experience.

It is in Section II., however, which deals with the subject of Refraction, that the superiority of the book is most clearly seen. Most English text-books, which are excellent in other respects, cannot be recommended as containing a good description of this subject, which is on the whole the most important in the whole range of ophthalmology.

Berry's book can be most highly recommended in this connection. Its treatment of refraction is complete in itself; and while it is sufficiently scientific and mathematical to satisfy those who possess a special knowledge on the subject, it is so clearly written and well expressed that it can be readily made practical use of by those whose knowledge of mathematics is very limited. Our author deals sensibly and practically with the clinical aspect of refractive errors and the use of lenses. His remarks on the etiology of Myopia are independent, and his remarks on prophylaxis and treatment indicate careful judgment.

In astigmatism our author regards retinoscopy as of great advantage; and it is just in this complicated error that the objective test is of such value. Berry admits the great difficulty of the direct method in astigmatism, but considers that it is excellent in spherical error, but difficult to apply to the amount of differentiation between the meridians. No one can deny the great merit of Javal's optometer; but a method by which the ophthalmoscope and ordinary lenses can be applied to the absolute estimation of astigmatism, and of spherical error at the same time, must be possessed of very great advantages.

The operations are clearly and fully described. It is always a difficult matter lucidly to describe a complicated operation, though easy to demonstrate it; but in this book description goes as far in making the various proceedings plain as is possible.

The operations more often performed in general practice, such as enucleation of the globe and slitting the canaliculus, are well given; so that in this respect, as well as in many others, this book will be of great use to general practitioners, especially in country practice. The operations generally performed only in ophthalmic practice also receive the fullest and most careful consideration.

Not only are the operations themselves well described, but their risks and the after-treatment necessary are all carefully given.

There is a very good account of the new methods of advancing the recti muscles in cases of strabismus; and the transplantation of mucous membrane from the mouth to the eye in symblepharon is described, though the latest proceeding, *i.e.* the transplantation of rabbit's conjunctiva, has not yet found a place in this work.

Mules' operation (evisceration with the insertion of a hollow globe) is mentioned. The author admits the very good result sometimes obtained, but recognises the liability to imperfect union of the conjunctiva over the artificial globe and its consequent tendency to drop out; but does not allude to the risk of sympathetic mischief, which must not be forgotten.

The great subject of discussion at the present time, cataract operation without iridectomy, is entered upon, though very much space is of course not given to it. Berry does not recommend it in all cases; but when the cataract is not only mature, but also soft, especially if cocaine produces very marked dilatation of the pupil, he suggests that the extraction should be performed without iridectomy.

The difficulties and accidents in cataract-extraction are well given, and the operator is instructed how to deal with them in a thoroughly practical way.

We are advised not to perform iridectomy *for glaucoma* with cocaine, as the author does not consider it acts sufficiently as a local anæsthetic in these cases; but in our experience of its use in some of the less acute forms it has seemed to act well. In iridectomy for artificial pupil merely, Berry recommends the use of Tyrrell's hook rather than iridectomy forceps; but where we have to remove a large piece of iris in glaucoma (though the absolute need for this is questioned), he advises the use of the usual forceps.

Altogether, we may derive the greatest instruction in practical matters relating to the performance of ophthalmic operations from our perusal of this part of the book, and rest assured that we have here presented to us the more modern methods of operative procedure.

